# Supplementary material for: Regulatory T cells control the dynamic and site-specific polarization of total CD4 T cells following Salmonella infection
Source: Mucosal Immunol. 2020 May 26;13(6):946–57. doi: 10.1038/s41385-020-0299-1 (PMC7567643; doi:10.1038/s41385-020-0299-1)
Supplement: Supplementary file 1 — Supplementary figures [file 41385_2020_299_MOESM1_ESM.pptx]

## Slide 1
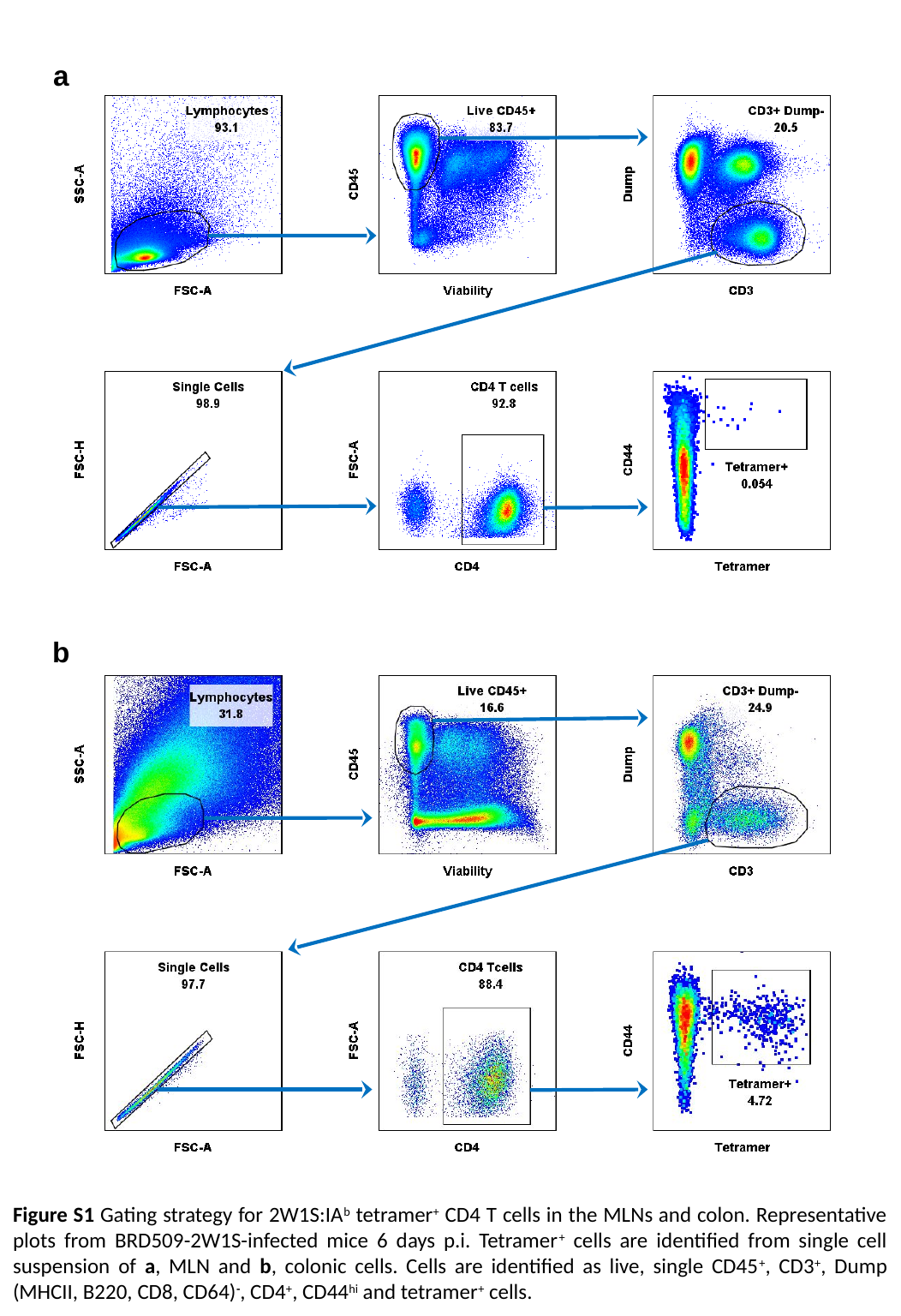

a
b
Figure S1 Gating strategy for 2W1S:IAb tetramer+ CD4 T cells in the MLNs and colon. Representative plots from BRD509-2W1S-infected mice 6 days p.i. Tetramer+ cells are identified from single cell suspension of a, MLN and b, colonic cells. Cells are identified as live, single CD45+, CD3+, Dump (MHCII, B220, CD8, CD64)-, CD4+, CD44hi and tetramer+ cells.

## Slide 2
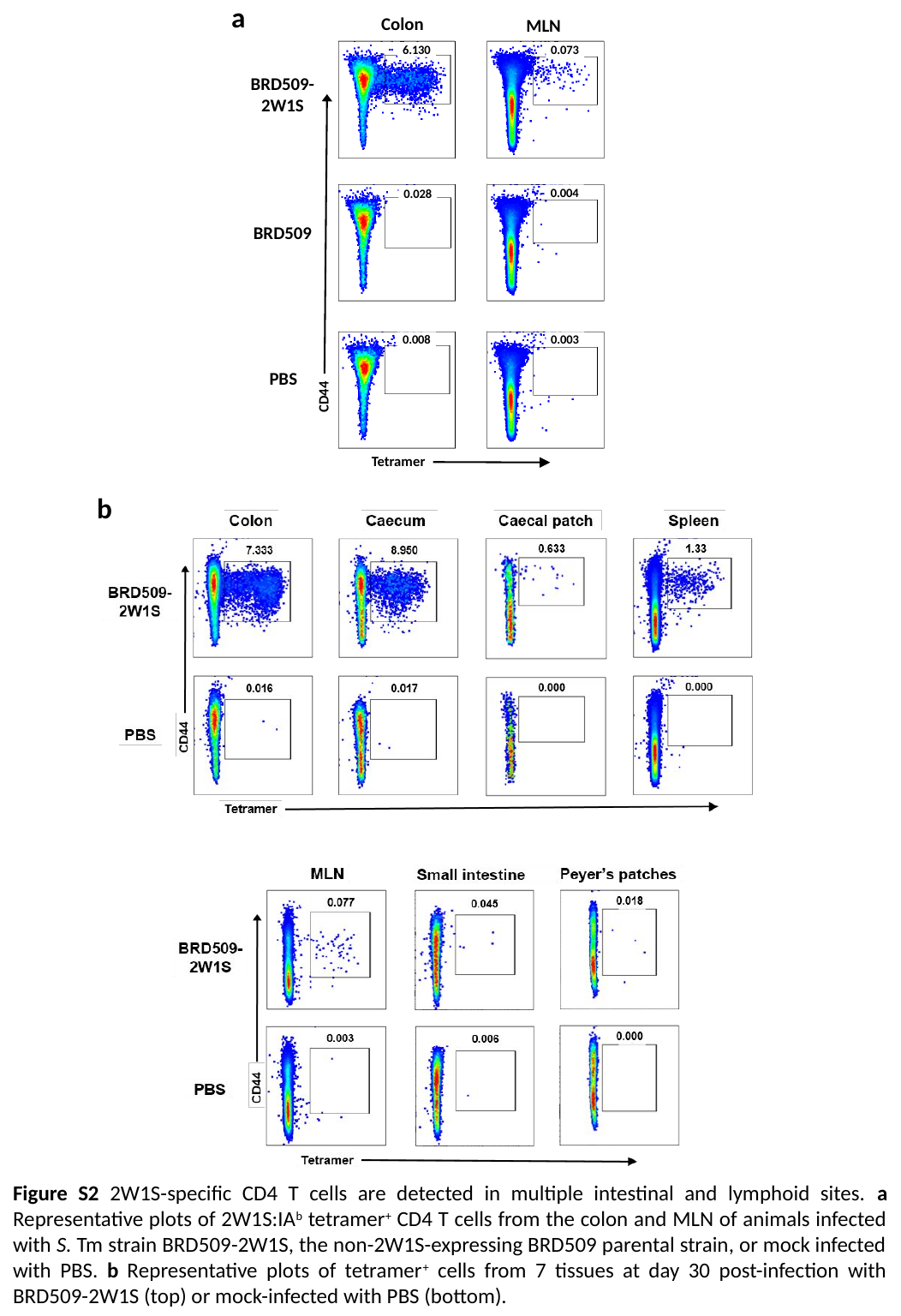

a
Colon
MLN
6.130
0.073
BRD509-
2W1S
0.004
0.028
BRD509
0.008
0.003
PBS
CD44
Tetramer
b
Figure S2 2W1S-specific CD4 T cells are detected in multiple intestinal and lymphoid sites. a Representative plots of 2W1S:IAb tetramer+ CD4 T cells from the colon and MLN of animals infected with S. Tm strain BRD509-2W1S, the non-2W1S-expressing BRD509 parental strain, or mock infected with PBS. b Representative plots of tetramer+ cells from 7 tissues at day 30 post-infection with BRD509-2W1S (top) or mock-infected with PBS (bottom).

## Slide 3
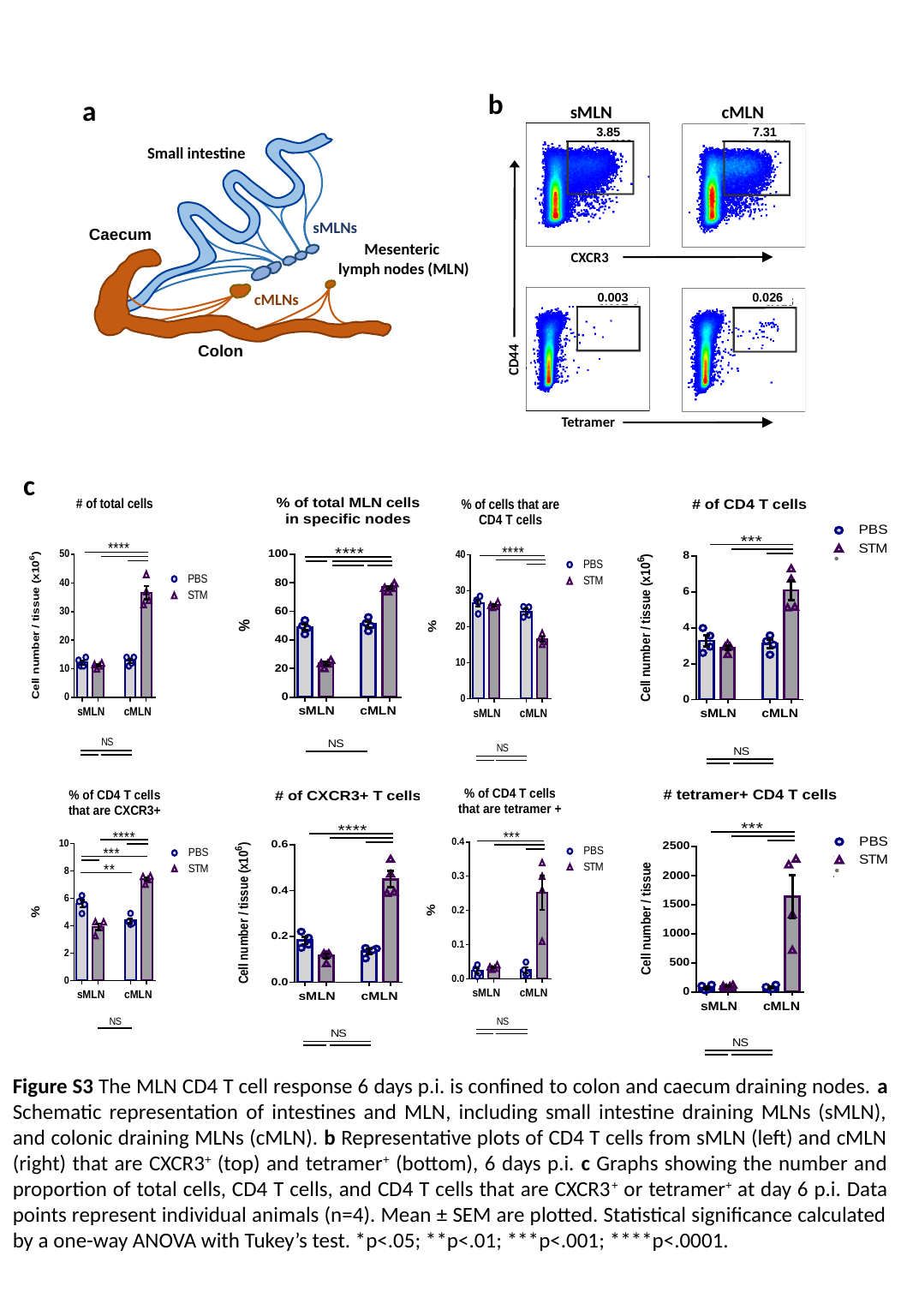

b
a
sMLN cMLN
CXCR3
CD44
Tetramer
Small intestine
sMLNs
Caecum
Mesenteric
lymph nodes (MLN)
cMLNs
Colon
7.31
3.85
0.003
0.026
c
.
.
Figure S3 The MLN CD4 T cell response 6 days p.i. is confined to colon and caecum draining nodes. a Schematic representation of intestines and MLN, including small intestine draining MLNs (sMLN), and colonic draining MLNs (cMLN). b Representative plots of CD4 T cells from sMLN (left) and cMLN (right) that are CXCR3+ (top) and tetramer+ (bottom), 6 days p.i. c Graphs showing the number and proportion of total cells, CD4 T cells, and CD4 T cells that are CXCR3+ or tetramer+ at day 6 p.i. Data points represent individual animals (n=4). Mean ± SEM are plotted. Statistical significance calculated by a one-way ANOVA with Tukey’s test. *p<.05; **p<.01; ***p<.001; ****p<.0001.

## Slide 4
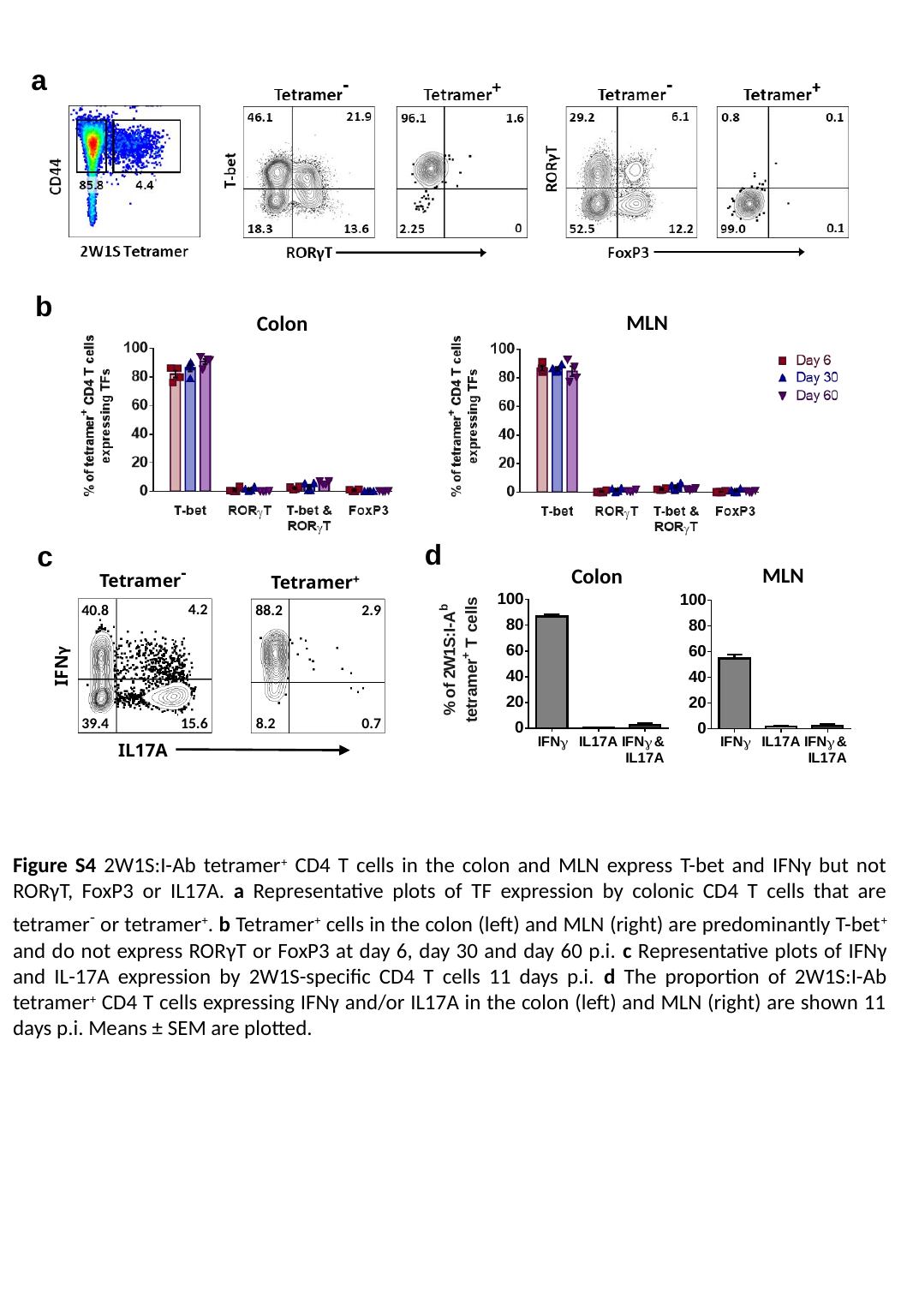

a
b
MLN
Colon
d
c
Tetramer-
MLN
Colon
Tetramer+
4.2
40.8
39.4
15.6
88.2
2.9
8.2
0.7
IFNγ
IL17A
Figure S4 2W1S:I-Ab tetramer+ CD4 T cells in the colon and MLN express T-bet and IFNγ but not RORγT, FoxP3 or IL17A. a Representative plots of TF expression by colonic CD4 T cells that are tetramer- or tetramer+. b Tetramer+ cells in the colon (left) and MLN (right) are predominantly T-bet+ and do not express RORγT or FoxP3 at day 6, day 30 and day 60 p.i. c Representative plots of IFNγ and IL-17A expression by 2W1S-specific CD4 T cells 11 days p.i. d The proportion of 2W1S:I-Ab tetramer+ CD4 T cells expressing IFNγ and/or IL17A in the colon (left) and MLN (right) are shown 11 days p.i. Means ± SEM are plotted.

## Slide 5
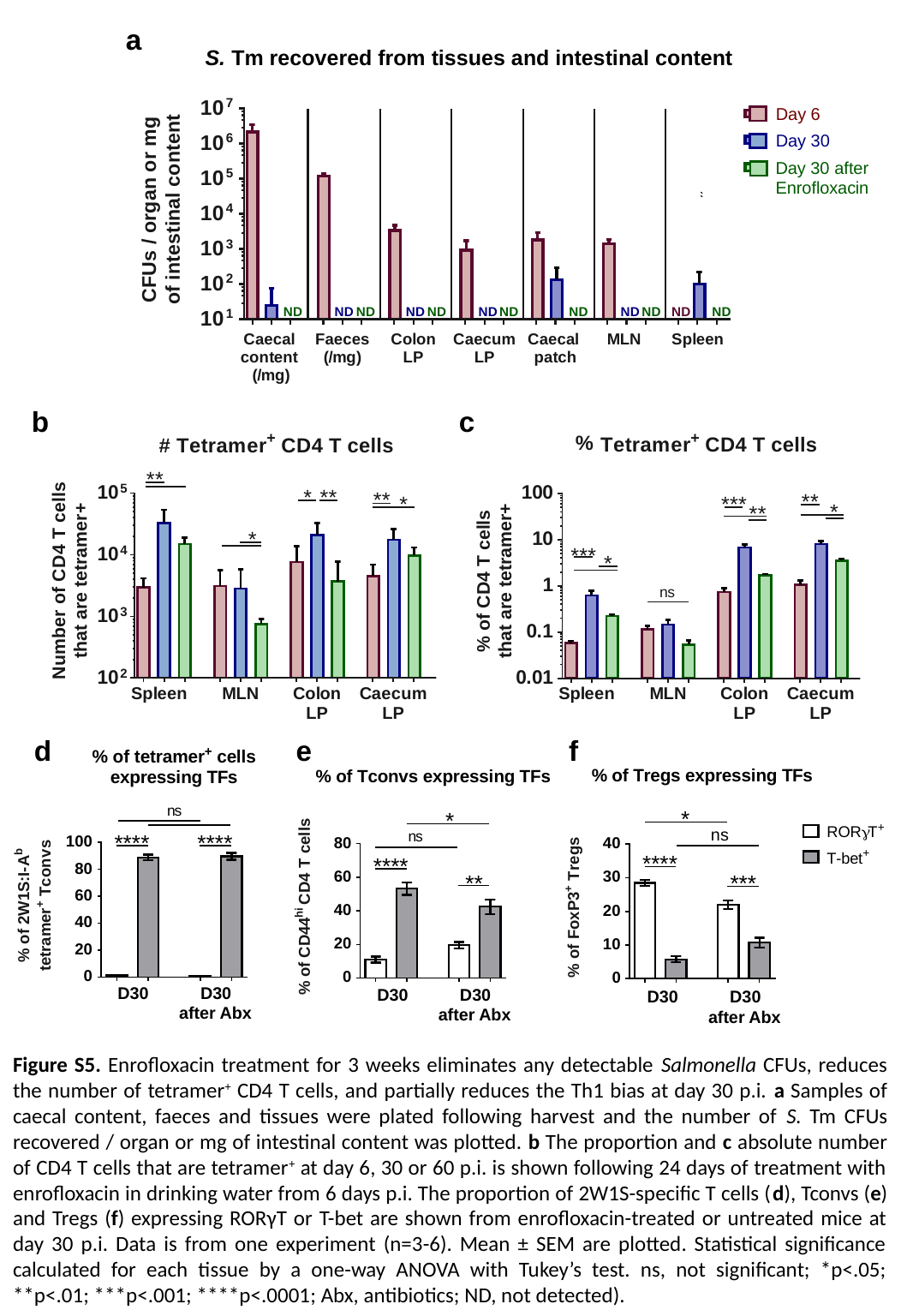

a
S. Tm recovered from tissues and intestinal content
b
c
f
e
d
D30 D30
 after Abx
D30 D30
 after Abx
D30 D30
 after Abx
Figure S5. Enrofloxacin treatment for 3 weeks eliminates any detectable Salmonella CFUs, reduces the number of tetramer+ CD4 T cells, and partially reduces the Th1 bias at day 30 p.i. a Samples of caecal content, faeces and tissues were plated following harvest and the number of S. Tm CFUs recovered / organ or mg of intestinal content was plotted. b The proportion and c absolute number of CD4 T cells that are tetramer+ at day 6, 30 or 60 p.i. is shown following 24 days of treatment with enrofloxacin in drinking water from 6 days p.i. The proportion of 2W1S-specific T cells (d), Tconvs (e) and Tregs (f) expressing RORγT or T-bet are shown from enrofloxacin-treated or untreated mice at day 30 p.i. Data is from one experiment (n=3-6). Mean ± SEM are plotted. Statistical significance calculated for each tissue by a one-way ANOVA with Tukey’s test. ns, not significant; *p<.05; **p<.01; ***p<.001; ****p<.0001; Abx, antibiotics; ND, not detected).

## Slide 6
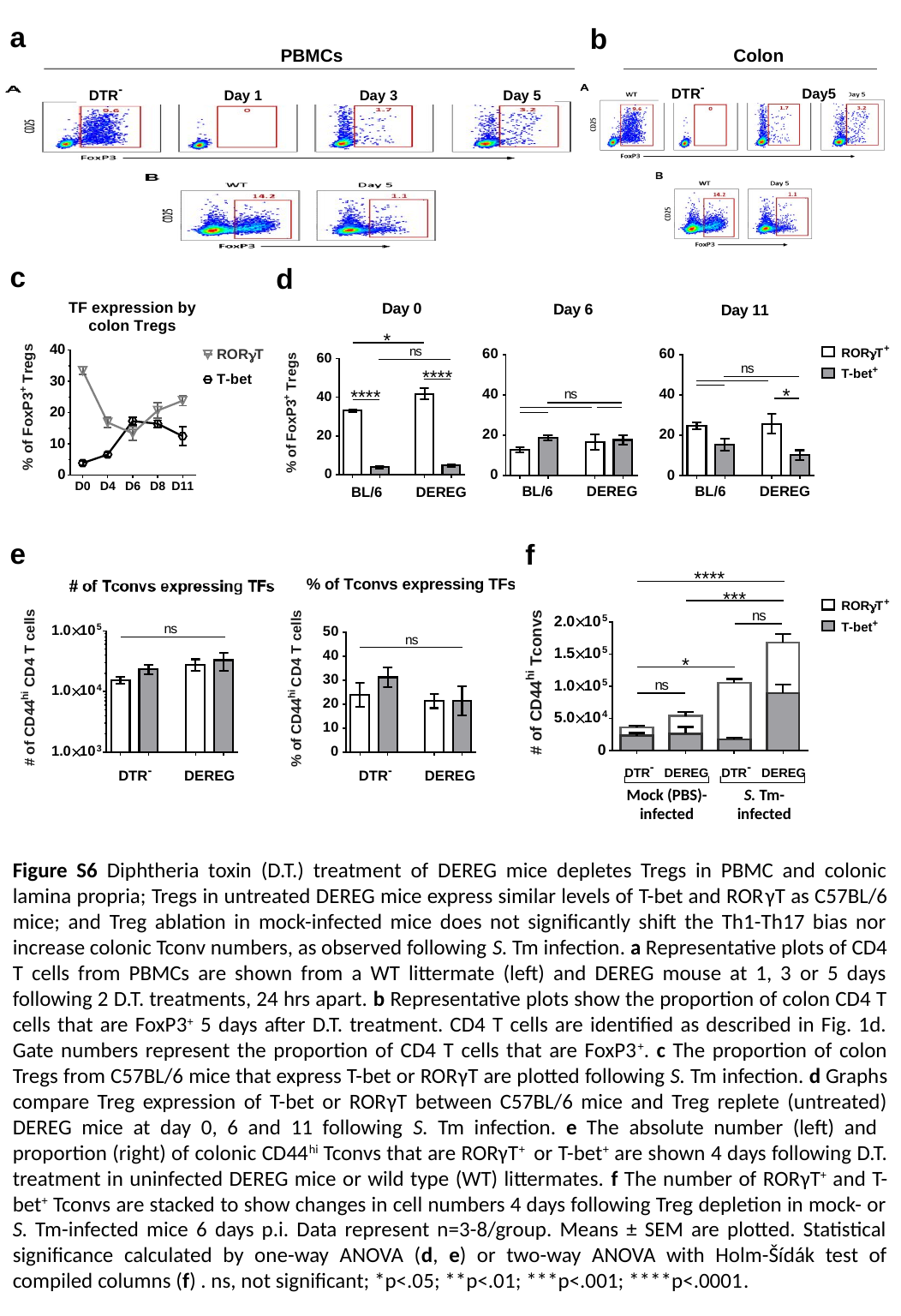

a
b
PBMCs Colon
DTR- Day5
DTR- Day 1 Day 3 Day 5
c
d
BL/6 DEREG
BL/6 DEREG
BL/6 DEREG
e
f
DTR- DEREG
DTR- DEREG DTR- DEREG
DTR- DEREG
Mock (PBS)-infected
S. Tm-
infected
Figure S6 Diphtheria toxin (D.T.) treatment of DEREG mice depletes Tregs in PBMC and colonic lamina propria; Tregs in untreated DEREG mice express similar levels of T-bet and RORγT as C57BL/6 mice; and Treg ablation in mock-infected mice does not significantly shift the Th1-Th17 bias nor increase colonic Tconv numbers, as observed following S. Tm infection. a Representative plots of CD4 T cells from PBMCs are shown from a WT littermate (left) and DEREG mouse at 1, 3 or 5 days following 2 D.T. treatments, 24 hrs apart. b Representative plots show the proportion of colon CD4 T cells that are FoxP3+ 5 days after D.T. treatment. CD4 T cells are identified as described in Fig. 1d. Gate numbers represent the proportion of CD4 T cells that are FoxP3+. c The proportion of colon Tregs from C57BL/6 mice that express T-bet or RORγT are plotted following S. Tm infection. d Graphs compare Treg expression of T-bet or RORγT between C57BL/6 mice and Treg replete (untreated) DEREG mice at day 0, 6 and 11 following S. Tm infection. e The absolute number (left) and proportion (right) of colonic CD44hi Tconvs that are RORγT+ or T-bet+ are shown 4 days following D.T. treatment in uninfected DEREG mice or wild type (WT) littermates. f The number of RORγT+ and T-bet+ Tconvs are stacked to show changes in cell numbers 4 days following Treg depletion in mock- or S. Tm-infected mice 6 days p.i. Data represent n=3-8/group. Means ± SEM are plotted. Statistical significance calculated by one-way ANOVA (d, e) or two-way ANOVA with Holm-Šídák test of compiled columns (f) . ns, not significant; *p<.05; **p<.01; ***p<.001; ****p<.0001.

## Slide 7
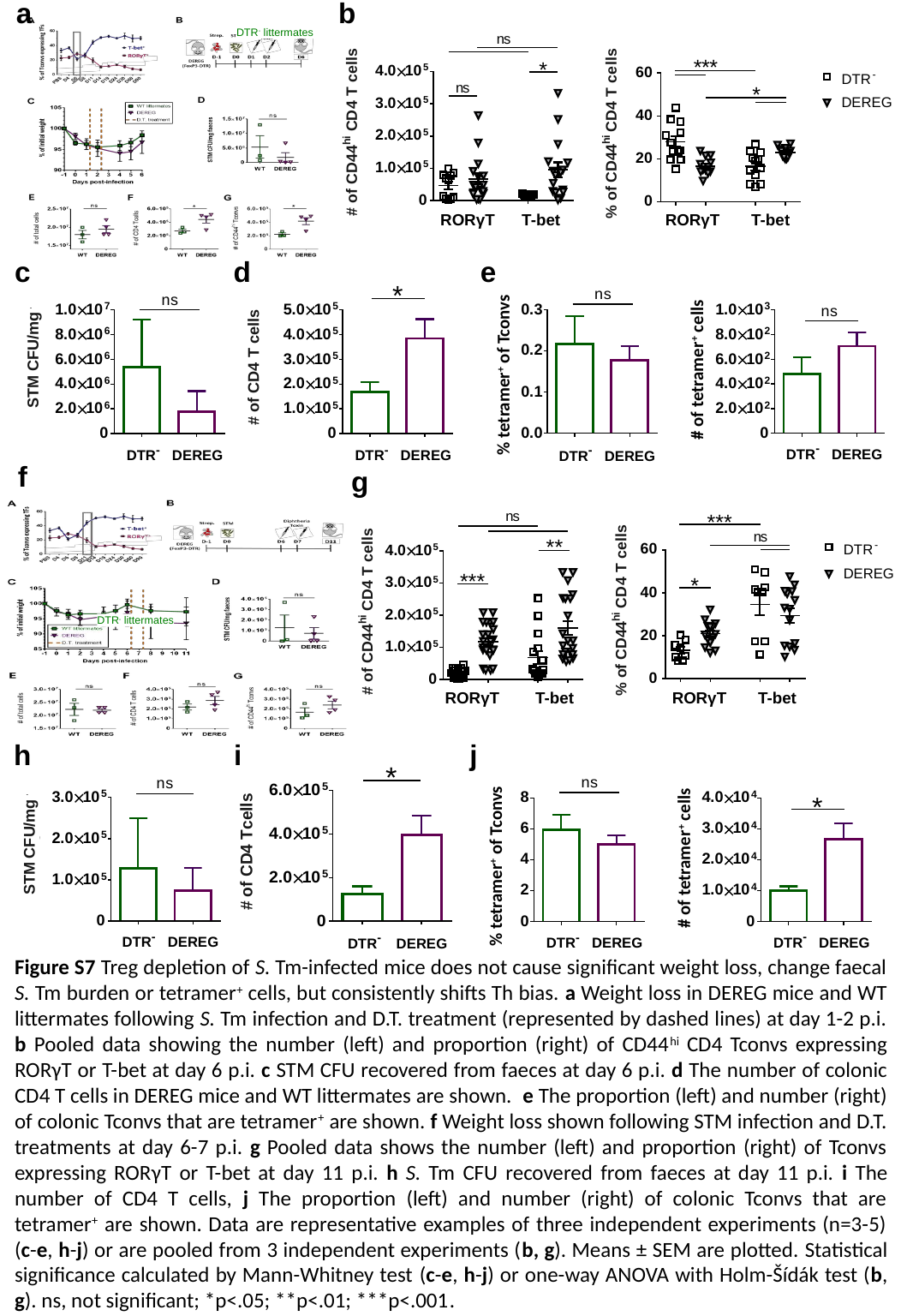

a
b
DTR- littermates
RORγT T-bet
RORγT T-bet
c
d
e
% tetramer+ of Tconvs
DTR- DEREG
DTR- DEREG
DTR- DEREG
DTR- DEREG
# of tetramer+ cells
f
g
DTR- littermates
RORγT T-bet
RORγT T-bet
h
i
j
% tetramer+ of Tconvs
DTR- DEREG
DTR- DEREG
DTR- DEREG
DTR- DEREG
# of tetramer+ cells
Figure S7 Treg depletion of S. Tm-infected mice does not cause significant weight loss, change faecal S. Tm burden or tetramer+ cells, but consistently shifts Th bias. a Weight loss in DEREG mice and WT littermates following S. Tm infection and D.T. treatment (represented by dashed lines) at day 1-2 p.i. b Pooled data showing the number (left) and proportion (right) of CD44hi CD4 Tconvs expressing RORγT or T-bet at day 6 p.i. c STM CFU recovered from faeces at day 6 p.i. d The number of colonic CD4 T cells in DEREG mice and WT littermates are shown. e The proportion (left) and number (right) of colonic Tconvs that are tetramer+ are shown. f Weight loss shown following STM infection and D.T. treatments at day 6-7 p.i. g Pooled data shows the number (left) and proportion (right) of Tconvs expressing RORγT or T-bet at day 11 p.i. h S. Tm CFU recovered from faeces at day 11 p.i. i The number of CD4 T cells, j The proportion (left) and number (right) of colonic Tconvs that are tetramer+ are shown. Data are representative examples of three independent experiments (n=3-5) (c-e, h-j) or are pooled from 3 independent experiments (b, g). Means ± SEM are plotted. Statistical significance calculated by Mann-Whitney test (c-e, h-j) or one-way ANOVA with Holm-Šídák test (b, g). ns, not significant; *p<.05; **p<.01; ***p<.001.

## Slide 8
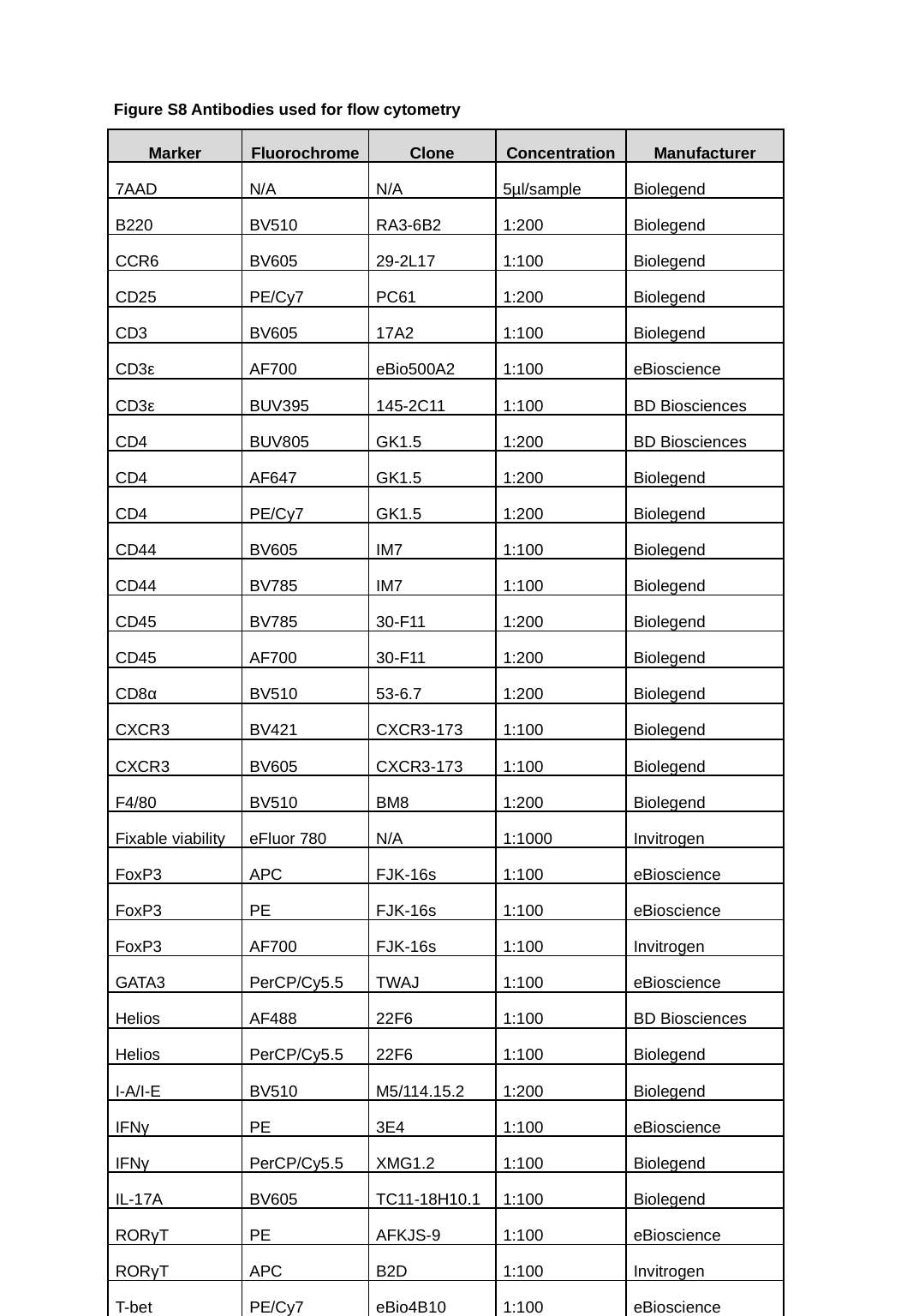

Figure S8 Antibodies used for flow cytometry
| Marker | Fluorochrome | Clone | Concentration | Manufacturer |
| --- | --- | --- | --- | --- |
| 7AAD | N/A | N/A | 5µl/sample | Biolegend |
| B220 | BV510 | RA3-6B2 | 1:200 | Biolegend |
| CCR6 | BV605 | 29-2L17 | 1:100 | Biolegend |
| CD25 | PE/Cy7 | PC61 | 1:200 | Biolegend |
| CD3 | BV605 | 17A2 | 1:100 | Biolegend |
| CD3ε | AF700 | eBio500A2 | 1:100 | eBioscience |
| CD3ε | BUV395 | 145-2C11 | 1:100 | BD Biosciences |
| CD4 | BUV805 | GK1.5 | 1:200 | BD Biosciences |
| CD4 | AF647 | GK1.5 | 1:200 | Biolegend |
| CD4 | PE/Cy7 | GK1.5 | 1:200 | Biolegend |
| CD44 | BV605 | IM7 | 1:100 | Biolegend |
| CD44 | BV785 | IM7 | 1:100 | Biolegend |
| CD45 | BV785 | 30-F11 | 1:200 | Biolegend |
| CD45 | AF700 | 30-F11 | 1:200 | Biolegend |
| CD8α | BV510 | 53-6.7 | 1:200 | Biolegend |
| CXCR3 | BV421 | CXCR3-173 | 1:100 | Biolegend |
| CXCR3 | BV605 | CXCR3-173 | 1:100 | Biolegend |
| F4/80 | BV510 | BM8 | 1:200 | Biolegend |
| Fixable viability | eFluor 780 | N/A | 1:1000 | Invitrogen |
| FoxP3 | APC | FJK-16s | 1:100 | eBioscience |
| FoxP3 | PE | FJK-16s | 1:100 | eBioscience |
| FoxP3 | AF700 | FJK-16s | 1:100 | Invitrogen |
| GATA3 | PerCP/Cy5.5 | TWAJ | 1:100 | eBioscience |
| Helios | AF488 | 22F6 | 1:100 | BD Biosciences |
| Helios | PerCP/Cy5.5 | 22F6 | 1:100 | Biolegend |
| I-A/I-E | BV510 | M5/114.15.2 | 1:200 | Biolegend |
| IFNγ | PE | 3E4 | 1:100 | eBioscience |
| IFNγ | PerCP/Cy5.5 | XMG1.2 | 1:100 | Biolegend |
| IL-17A | BV605 | TC11-18H10.1 | 1:100 | Biolegend |
| RORγT | PE | AFKJS-9 | 1:100 | eBioscience |
| RORγT | APC | B2D | 1:100 | Invitrogen |
| T-bet | PE/Cy7 | eBio4B10 | 1:100 | eBioscience |
